# Real-Time Indoor Scene Description for the Visually Impaired Using Autoencoder Fusion Strategies with Visible Cameras

**DOI:** 10.3390/s17112641

**Published:** 2017-11-16

**Authors:** Salim Malek, Farid Melgani, Mohamed Lamine Mekhalfi, Yakoub Bazi

**Affiliations:** 1 Department of Information Engineering and Computer Science, University of Trento, Via Sommarive 9, I-38123 Trento, Italy; salim.malek@unitn.it (S.M.); mohamed.mekhalfi@alumni.unitn.it (M.L.M.); 2 College of Computer and Information Sciences, King Saud University, Riyadh 11543, Saudi Arabia; ybazi@ksu.edu.sa

**Keywords:** assistive technologies, visible cameras, visually impaired (VI) people, coarse scene description, multiobject recognition, deep learning, feature fusion, image representation

## Abstract

This paper describes three coarse image description strategies, which are meant to promote a rough perception of surrounding objects for visually impaired individuals, with application to indoor spaces. The described algorithms operate on images (grabbed by the user, by means of a chest-mounted camera), and provide in output a list of objects that likely exist in his context across the indoor scene. In this regard, first, different colour, texture, and shape-based feature extractors are generated, followed by a feature learning step by means of AutoEncoder (AE) models. Second, the produced features are fused and fed into a multilabel classifier in order to list the potential objects. The conducted experiments point out that fusing a set of AE-learned features scores higher classification rates with respect to using the features individually. Furthermore, with respect to reference works, our method: (i) yields higher classification accuracies, and (ii) runs (at least four times) faster, which enables a potential full real-time application.

## 1. Introduction

Strolling around, adjusting the walking pace and bodily balance, perceiving nearby or remote objects and estimating their depth, are all effortless acts for a well-sighted person. That is, however, hardly doable for other portions in society, such as individuals with certain cases of handicap, or visual impairment, which may require different forms of substantial training, and in many situations external physical and/or verbal intervention as to ease their mobility. In dealing with that, numerous attempts at different governmental, institutional, as well as societal spheres have been taking place.

One assistive line, ought to be undertaken by various research institutions, is the providence of either technological designs or end-user products that can help bridging the gap between the conditions being experienced by such disabled people and their expectations. As per the physically handicapped category, a well-established amount of rehabilitation (particularly robotic-based) layouts has been developed so far. However, when it comes to blindness rehabilitation technologies, relatively fewer attentions have been drawn in the relevant literature. As a side note, depending upon the severity of sight loss, vision disability is an umbrella term that encompasses a wide range of progressively inclusive cases, since it could be diagnosed as a: (i) mild impairment, (ii) middle-range impairment, (iii) severe impairment, and ends up to the unfortunate (iv) full blindness. Full sight loss is therefore a serious disability that entails far-reaching ramifications, as it blocks in many cases, the affected individual from conducting his/her daily routines smoothly.

In order to enable the visually disabled persons to move around more easily, several contributions have been proposed in the literature, which are commonly referred to as Electronic Travel Aids (ETAs). By and large, the current ETA methodologies can be identified according to two distinct but complementary aspects, namely: (i) mobility and navigation assistance, that undertakes as a goal assisting visually disabled people to autonomously walk around with the possibility to sense nearby obstacles, and avoid potential collisions thereby, and (ii) object recognition, whose underlying motive is to aid them recognize objects.

Regarding the mobility and obstacle avoidance part, a reasonable amount of works has been put forth thus far. Pundlik et al. [1], for instance, developed a collision detection approach based on a body-mounted camera for visually impaired (VI) people. They proceed by computing the sparse optical flow in the acquired videos and make use of a gyroscopic sensor to estimate the camera rotation. The collision risk is then estimated from the motion estimates. In another work, Balakrishnan et al. [2], presented a system to detect obstacles. The blind individual carries two small cameras mounted on sunglasses. From the captured pair of images, the disparity map is generated and the distances of the objects to the cameras are estimated, which allow for a further decision whether the objects lying ahead of the user make a potential threat. Another navigation aid, named the guide cane, was introduced in [3], which comprises a wheeled housing supplied with a set of ultrasonic sensors, a handle to guide the cane through, and a processing core that processes the ultrasonic signals emitted by the sensors as to infer whether an object is present along the walking path. The concept of the ultrasonic sensors is that they simultaneously emit beams of signals, which in case of obstacles if any, are reflected back. The distance to the obstacles is then deduced based on the time lapse between emission and reflection (commonly termed as time of flight—TOF). The same concept was adopted in [4], where the sensors are placed on a wearable belt instead. Another similar work was put forth in [5]. In this work, the sensors were placed on the shoulders of the user as well as on a guide cane. Another unique contribution proposes exploiting electromagnetic signals instead of ultrasonic ones by using a widespread antenna [6]. However, the capacity of the proposed prototype is limited to 3 m ahead of the user. Having a close look at the literature, it emerges clearly that TOF-based concepts have often been employed and exhibited promising outcomes. The apparent downsides of such methodologies, however, are mainly confined to the dimensions as well as weight of the developed prototypes on the one hand, which may compromise the user’s convenience, and the demanding power consumption (i.e., constant emission/reception of ultrasonic signals) on the other hand.

Regarding the object recognition aspect, introspectively far less contributions can be observed. This might be traced back to the reason that object recognition for the blind might be a harder task to fulfil as compared to navigation and object avoidance. In other words, mobility and object avoidance does not pay attention to the kind of potential objects but to their presence instead, whilst object recognition emphasises on the nature of the nearby objects (i.e., not only their existence). Furthermore, recognizing objects, in camera-shot images, might come at the cost of several challenges such as rotation, scale, and illumination variations, notwithstanding the necessity to carry out such task in a brief time lapse. Nevertheless, different computer-vision techniques have been tailored to tackle this issue. In [7], for instance, a food product recognition system in shopping spaces was proposed. It relies on detecting and recognizing the QR codes of food items by means of a portable camera. Another work considers detecting and recognizing bus line numbers for the VI [8]. Banknote recognition has also been addressed in [9]. Staircases, doors, and indoor signage detection/ recognition have been considered in [10,11,12]. In [13], the authors developed a prototype composed of ultrasonic sensors and a video camera, which is embedded in a smartphone for a real-time obstacle detection and classification. They first extract FAST feature points from the image and track them with a multiscale Lucas-Kanade algorithm. Then, in the classification phase, a Support Vector Machine was used to detect one of the four objects defined a priori. Consequently, it can be observed that the scarce amount of works that have been devoted to assisted object recognition for the VI so far, emphasize on detecting/recognizing single classes of objects. On this point, it is believed that extending the process into a multiobject recognition is prone to provide a richer description for the VI people.

Subsequently, posing the case of multiobject recognition in general, the mainstream research line suggests designing as many models as the number of objects of interest and then run those learned models on a given query image as to discern its potential object list. Such paradigm could be of notable efficiency, but it is achievable at the cost of prohibitively large processing overheads, which is not a wise choice if undertaken in the context of assistive recognition for the VI people. Departing from this limitation, Mekhalfi et al. [14] introduced a novel approach called coarse description, which operates on portable camera-grabbed images by listing the objects existing in a given nearby indoor spot, irrespective of their location in the indoor space. Precisely, they proposed Scale Invariant Feature Transform (SIFT), Bag of Words (BOW), and Principal Component Analysis (PCA) strategies as a means of image representation. For the sake of furthering the performance of their coarse image description, they suggested another scheme, which exploits Compressive Sensing (CS) theory for image representation and a semantic similarity metric for image likelihood estimation through a bunch of learned Gaussian Process Regression (GPR) models, and concluded that a trade-off between reasonable recognition rates and low processing times can be maintained [15].

In this paper, we propose a new method to describe the surrounding environment for a VI person in real-time. We use Local Binary Pattern (LBP) technique, Histogram of Oriented Gradient (HOG) and BOW to describe coarsely the content of the image acquired via an optical camera. In order to improve the state of the art results and deal properly with runtime, we propose to use a deep learning approach, in particular an Auto Encoder Neural Network (AE), to create a new high-level feature representation from the previous low-level features (HOG, BoW and LBP). Once generated, the new feature vectors are fed into a logistic regression layer using a multi-labelling strategy as to draw the objects present in the image of concern. This work is a part of a project to guide a VI person in an indoor environment. As validated by the experimental setup, tangible recognition gains and significant speedups have been scored with respect to recent works.

In what follows, Section 2 recalls the coarse scene description in brief. Section 3 provides short but self-contained conceptual backgrounds of the different methodologies employed for image representation. Section 4 outlines the image multi-labeling pipeline, which is meant for coarse description. In Section 5, we quantify the recognition rates and the processing time and discuss the different pros and cons of the proposed method in the context of indoor scene description. At last, we brief conclusions and draw potential rooms for improvements.

## 2. Coarse Description

**Figure 1:**
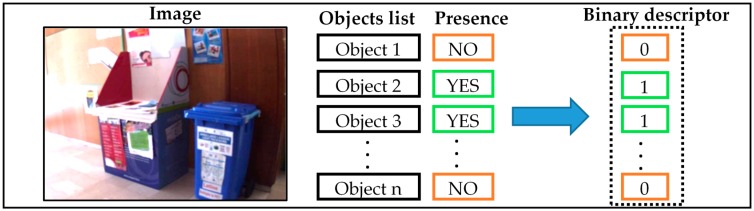
Binary descriptor construction for a training image.

As mentioned earlier, the key idea is to sacrifice the objects’ coordinates across the indoor space with the processing requirements, so as to render the recognition process faster yet more convenient for (at least near) real-time scenarios. In this respect, a coarse description of images captured in an indoor environment is adopted. The principle of this approach consists in checking the presence/absence of different objects, which were determined a priori, and turns out to convey the list of the objects that are most likely present in the scene. This approach is based on the multi-labelling strategy by creating a binary vector (vector of labels). This vector, as shown in Figure 1, indicates which objects are present/absent in its corresponding image. In the training phase, a set of images are captured from the indoor environment and stored with their binary vector. In the classification phase, the proposed method gives in output a multi-label vector referring to the list of existing objects in the scene. This new representation aims to enhance the perception of the VI individual regarding the surrounding environment.

## 3. Tools and Concepts

Let us consider a colour image X acquired by a portable digital camera in an indoor environment. Due to several inherent properties of the images, such as illumination, rotation and scale changes, the images cannot be used in their raw form but need to be transformed into an adequate feature space that is able to capture the spatial as well as the spectral variations. Such objective can normally be addressed from three perspectives, namely: (i) shape information, (ii) colour information, and (iii) textural changes. On this point, adopting one feature modality while omitting the others may drop the robustness of the classification algorithm being developed. We therefore resort to a more efficient representation, by making use of all three feature modalities. Precisely, we opt for reputed feature extractors. The first one is the HOG [16] to feature the different shapes distributed over the images. The second one is the BOW [17] based on colour information of the different chromatic channels (BOW_RGB). Finally, the LBP technique in order to express the textural behaviour of the images. As a matter of fact, all the mentioned features can yield interesting results, and this has been documented by previous works, mainly related to object, texture recognition, biometrics as well as remote sensing. In order to further boost their representativeness, we also put forth a feature learning scheme that maps the original feature vectors (derived by means of either feature type mentioned above) onto another lower/higher feature space that offers a better feature representation capability. A well-established feature learning model is the Stacked Auto-encoder (SAE) neural network, or simply Autoencoder (AE), which constructs a model learned on features pertaining to training images, and then applies it on a given image in order to produce a final image representation.

**Figure 2:**
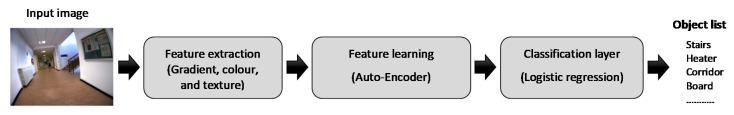
Pipeline of the feature learning-based image multilabeling scheme.

The final step of the proposed image multilabeling method is the classification of the generated features. This step is performed by appending a logistic regression layer (LRL) to the top of the network. The general diagram of the multilabeling procedure is depicted in Figure 2. The following subsections are dedicated to provide basic elaborations of the feature extraction and learning methodologies.

### 3.1. Histogram of Oriented Gradient

The HOG was initially aimed at pedestrian detection [16]. Soon later, it was utilized in other applications ranging from object recognition and tracking to remote sensing [18,19]. The basic idea of the HOG is to gather the gradient variations across a given image. Basically, this can be done by dividing the image into adjacent small-sized areas, called cells, and calculating the histograms of the magnitudes/directions of the gradient for the pixels within the cell. Each pixel of the cell is then assigned to one of the bins of the histogram, according to the orientation of the gradient at this point. This assignment is weighted by the gradient of the intensity at that point. Histograms are uniform from either 0 to 180° (unsigned case) or from 0 to 360° (signed case). Dalal and Triggs [16] point out that a fine quantization of the histogram is needed, and they get their best results with a 9-bin histogram. The combination of the computed histograms then forms the final HOG descriptor.

### 3.2. Bag of Visual Words

The BOW is a very popular model in the general computer vision literature. It is usually adopted for its notable property of promoting a concise but rich representation of a generic image. BOW signatures are generally reproduced from a certain feature space of the images, it can be the spectral intensities or alternatively keypoint-based descriptors derived from the images. The BOW is opted for in our work in order to produce a compact representation of the colour attributes of an image. We therefore depart from the chromatic (Red, Green, and Blue channels) values of the images. At first, a basis commonly referred to as codebook is established by gathering all the spectral features of the training images into a matrix. Afterwards, we apply a clustering technique i.e., the K-means clustering, on the built matrix to narrow down its size, which points out a small-sized basis (codebook). Next, the occurrences of the elements (words) of the codebook are observed in the chromatic space of a given image, which turns out to generate a compact histogram whose length equals to the number of the codebook’s words. For a more detailed explanation, the reader is referred to [14,17].

### 3.3. Local Binary Pattern (LBP)

Texture is a very important information that can play a key-role in characterizing images and their objects. One of the most popular techniques in this regard is the Local Binary Pattern (LBP) which is a multiresolution, gray-scale, and rotation invariant texture representation. It was first proposed by Ojala et al. [20] and then improved by Guo et al. [21] who introduced a variant called Completed Local Binary Pattern (CLBP), followed by many other variants. The following part gives a brief review about the basic LBP operator. Given a pixel in the image z(*u*, *v*), its LBP code is computed by comparing its intensity value to the values of its local neighbours:

(1) $$LBP_{P,R}\left(u,v\right)=\overset{P-1}{\underset{p=0}{\sum }}H\left(z\left(u,v\right)-z\left(u_{p},v_{p}\right)\right)2^{p}$$

where $z\left(u_{p},v_{p}\right)$ is the grey value of its *p*th neighbouring pixel, *P* is the total number of neighbours, *R* is the radius of the neighbourhood and *H*(∙) is the Heaviside step function.

The coordinates of the neighbour $z\left(u_{p},v_{p}\right)$ are: $u_{p}=u+R\cos\left(\frac{2\pi p}{P}\right)\mathrm{and}\;v_{p}=v-R\sin\left(\frac{2\pi p}{P}\right)$. If the neighbors do not fall at integer coordinates, the pixel value is estimated by interpolation. Once the LBP label is constructed for every pixel $z\left(u,v\right)\in {ℛ}_{i}$, a histogram is generated to represent the texture region as follows:

(2) $$Hist\left(k\right)=\underset{u}{\sum }\underset{v}{\sum }\delta \left(LBP_{P,R}\left(u,v\right),k\right),\;k\in \left[0,\;N_{bins}\right]$$

where $N_{bins}$ is the number of bins and δ is the delta function.

In order to give more robustness for LBP and make it more discriminative, a similar strategy to the HOG method is applied. First, the image is divided into cells and the LBP is calculated for each cell. Then, the computed LBPs are combined to form the final LBP descriptor.

### 3.4. Autoencoder Networks (AE)

The AE is at the basis a neural network architecture characterized by one hidden layer. It has then three layers, one visible layer of size n, one hidden layer of d nodes and one reconstruction layer with n nodes. Let $x\in {ℛ}^{n}$ be the input vector, $h\in {ℛ}^{n}\;$ the output of the hidden layer and $\hat{x}\in {ℛ}^{n}$ the output of the AE (reconstruction of x). *d* can be inferior or superior to *n*. In the former case (i.e., *d* < *n*), the AE performs feature reduction. In the latter case, however, it performs an over-complete representation [22].

**Figure 3:**
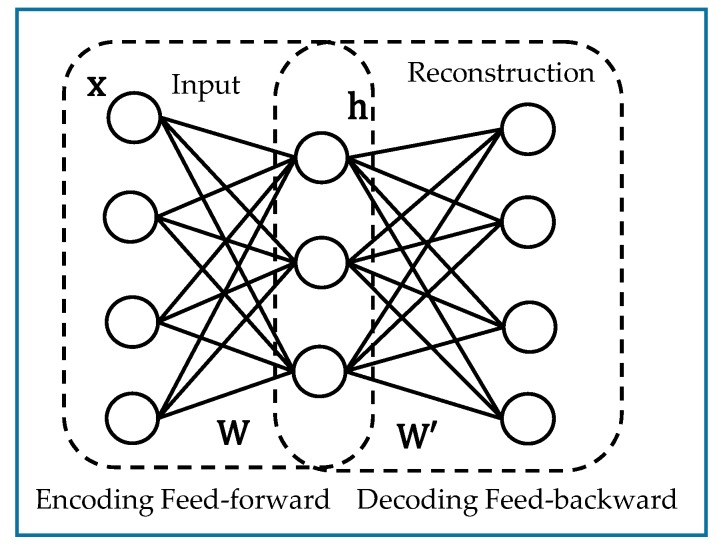
One layer architecture of an AE.

As can be shown in Figure 3, the output of the hidden and reconstruction layers can be calculated using the following equations:

(3) h=f(Wx+b)

(4) $$\hat{x}=f\left(W\prime h+b\prime \right)$$

where *f*(·) is a non-linear activation function, W and b are the *d* × *n* weight matrix and the bias vector of dimension *d* of the encoding, and W′ and b′ are the *n* × *d* weight matrix and the bias vector of dimension n of the decoding part.

The parameters (W, W′, b and b′) can be estimated by minimizing a cost function through a back-propagation algorithm [23]:

(5) $$\underset{W,\;W^{\prime },\;b,\;b^{\prime }}{\mathrm{argmin}}[L\left(x,\hat{x}\right)]$$

The loss function $L\left(x,\hat{x}\right)$ adopted in this work is the squared error i.e., ${\left\|x-\hat{x}\right\|}^{2}$. After finding the optimal values of weights and biases, we proceed by removing the last layer (i.e., reconstruction) with its corresponding parameters (W′ and b′). The layer ‘h’ therefore contains a new feature representation, which can be directly used as inputs into a classifier, or alternatively fed into another higher layer to generate deeper features.

In our case, we add a multinomial logistic regression layer (LRL), known also as softmax classifier, at the end of the encoding part to classify the produced feature representations. The choice of using a LRL is justified by its simplicity and the fact that it does not require any parameter tuning. The LRL is trained by adopting the output of the encoding part (the new feature representation) as input, and the corresponding binary vector as target output.

## 4. Feature Fusion

**Figure 4:**
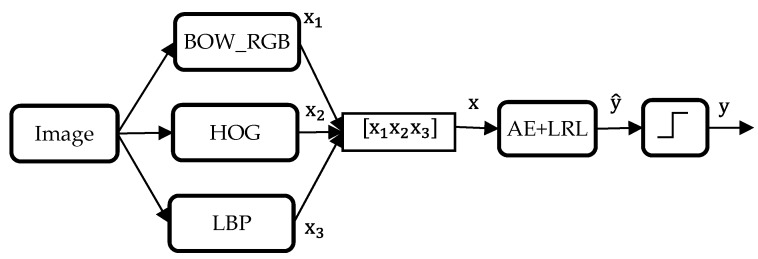
Diagram of the first fusion strategy (Fusion 1) based on a low-level feature aggregation.

As described so far, three types of features are made use of in this work (HOG, BOW_RGB, and LBP). In order to further improve the classification efficiency, we propose three distinct feature fusion schemes. The first one is a stacked fusion, which consists of extracting the three feature vectors from a given image and then stack them up to form a global feature vector. This latter is injected as an input to an AE topped by a logistic regression layer (LRL). The general diagram of the stacked fusion is observed in Figure 4.

**Figure 5:**
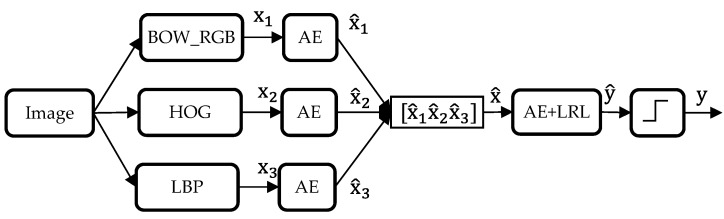
Diagram of the second fusion strategy (Fusion 2) based on a AE induced-level aggregation.

The second technique is a parallel fusion, as shown in Figure 5, which proceeds by feeding each type of feature into an individual AE model, followed by concatenating the learned features to form a single vector. This latter is set as input to another AE model that is connected to a LRL that outputs the final classification results. It is worth to mention that the two blocks composing the autoencoders are trained separately.

**Figure 6:**
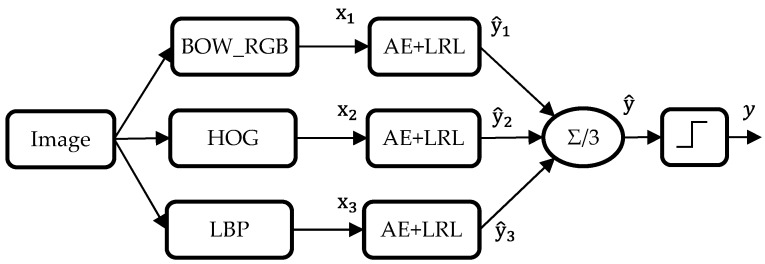
Diagram of the third fusion strategy (Fusion 3) based on a decision-level aggregation.

The third method is based on a linear sum of the individual decisions of the three types of features. In other words, each feature vector is fed into a separate AE model topped by a LRL. The outputs of each LRL are then averaged to come down to a single real-valued output, which is subsequently thresholded to force its values to either one or zero. Figure 6 gives an illustration of the fusion procedure.

## 5. Experimental Results

### 5.1. Description of the Wearable System

**Figure 7:**
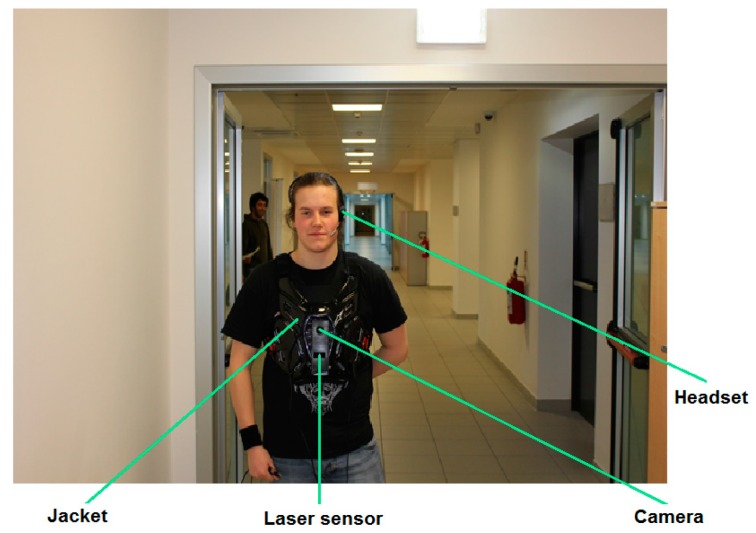
View of the wearable prototype with its main components.

The developed method is part of a complete prototype which is composed of two parts. The first part is the guidance system, which is responsible of guiding a visually impaired person across an indoor environment from an initial point to a desired destination taking into account the avoidance of the different static and/or dynamic obstacles. The second part is the recognition system, which is meant to describe the indoor site for the blind individual to give him better ability to sense the nearby surrounding environment by providing him with a list of existing objects. Regarding the hardware, the wearable system is composed of a laser range finder for detecting and determining objects distance to the user, a portable CMOS camera model UI-1240LE-C-HQ (IDS Imaging Development Systems, Germany) equipped with a LM4NCL lens (KOWA, Japan), a portable processing unit which can be a laptop, a tablet or a smartphone and a headset for voice input and audio output. The user controls the system by giving vocal instructions (i.e., specific keywords) via a microphone and receives information (e.g., list of objects) vocally synthesized through the earphone. All the hardware is mounted on a wearable jacket as can be seen on Figure 7. The design of the entire prototype was performed by taking into consideration the feedbacks we received from VI persons, in particular regarding interfacing and exploitation.

### 5.2. Dataset Description

The set of images used in this work was acquired by means the CMOS UI-1240LE-C-HQ camera, with KOWA LM4NCL lens, which is carried by a wearable lightweight shield. The images were shot at two different indoor spaces within the faculty of science at the University of Trento (Italy). The size of each image is 640 × 480. The first ensemble amounts to a total of 130 images, which was divided into 58 training and 72 testing images. The second set accounts for 131 images, split up into 61 training images, and 70 for testing purposes. It is noteworthy that the training images for both datasets were selected in such a way to cover all the predefined objects in the considered indoor environments. To this end, we have selected the objects deemed to be the most important ones in the considered indoor spaces. Regarding the first dataset, 15 objects were considered as follows: ‘External Window’, ‘Board’, ‘Table’, ‘External Door’, ‘Stair Door’, ‘Access Control Reader’, ‘Office’, ‘Pillar’, ‘Display Screen’, ‘People’, ‘ATM’, ‘Chairs’, ‘Bins’, ‘Internal Door’, and ‘Elevator’. Whereas, for the second dataset, the list was the following: ‘Stairs’, ‘Heater’, ‘Corridor’, ‘Board’, ‘Laboratories’, ‘Bins’, ‘Office’, ‘People’, ‘Pillar’, ‘Elevator’, ‘Reception’, ‘Chairs’, ‘Self Service’, ‘External Door’, and ‘Display Screen’.

### 5.3. Evaluation Metrics and Parameter Setting

For evaluation purposes, we use the well-known sensitivity (SEN) and specificity (SPE) measures:

(6) $$\mathrm{SEN}=\frac{True\;Positive}{True\;Positive+False\;Negative}$$

(7) $$\mathrm{SPE}=\frac{True\;Negative}{True\;Negative+False\;Positive}$$

The sensitivity expresses the classification rate of real positive cases i.e., the efficiency of the algorithm towards detecting existing objects. The specificity, on the other hand, underlines the tendency of the algorithm to detect the true negatives i.e., the non-existing objects. We also propose to compute the average of the two earlier measures as follows:

(8) $$\mathrm{AVG}=\frac{\mathrm{SEN}+\mathrm{SPE}}{2}$$

We set the parameters of the three feature extractors as follows.

- For HOG features, we set the number of bins to 9 and the size of the cells to 80, which gives a HOG feature vector of size 1260 (recall that the size of the image is 640 × 480).
- Regarding the BOW_RGB, the number of centroids i.e., ‘K’ of the K-means clustering is set to 200, which was observed as the best choice among other options.
- For the LBP, we set *R* = 1, *P* = 8, and size of the cells = 80, which produces a LBP feature vector of length 480 bins.

**Figure 8:**
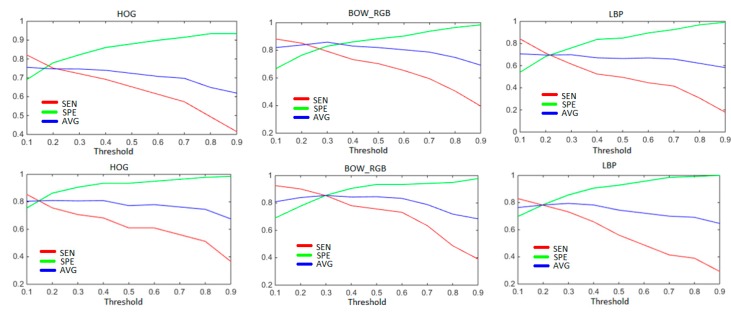
Impact of the threshold value on the classification rates using the three feature types. Upper row for Dataset 1, bottom row for Dataset 2.

In input layer, all values are normalised between 0 and 1. Regarding output, In fact, the LRLs point out real values. In order to force them to ones or zeroes, a thresholding must take place. Therefore, to determine the most convenient threshold, we applied a 5-folds cross validation technique on the training dataset, with 4 folds chosen randomly as training and the last one as test. The threshold values range from 0.1 to 0.9 with a step of 0.1. We repeated the experiments 5 times, each time with different random permutation. The results presented in Figure 8, refer to the average of the sensitivity and the specificity (along with their average) by means of the first fusion method. By observing those figures, SEN and SPE exhibit opposite behaviours as the threshold value increases. On average between SEN and SPE, a threshold of 0.3 stands out as the best option, which will be adopted in the remaining experiments.

### 5.4. Results

We first report the results pointed out by using the three types of features individually. We tried many configuration by changing the size of the hidden layer from 100 to 1000 nodes by a step of 100. Ultimately, 300 nodes turned out to be the best choice for all the features. It can be observed from Table 1 that on Dataset 1, the three features perform closely, with a slight improvement being noticed with BoW_RGB. On dataset 2, however, the BoW_RGB outperforms, by far, the remaining two, which was expected beforehand as this dataset particularly manifests richer colour information than the former one. Nevertheless, the yielded rates are quite reasonable taking into account the relatively large number of objects considered in this work, besides other challenges such as scale and orientation changes.

Coming to the fusion scenarios, an interesting point to initiate with is the determination of the optimal size of the hidden layer (i.e., number of hidden units). Different architectures have been explored. Precisely, we tried out values within the range of 100–1000 with a step of 100. Ultimately, the first fusion technique ended up having 900 neurons as an optimal choice, whilst the remaining two strategies pointed out their best at 500 and 300, respectively. It is to note that all the values within 100–1000 have pointed out nearby performances. The earlier optimal parameters will therefore be adopted in what follows.

**Figure 9:**
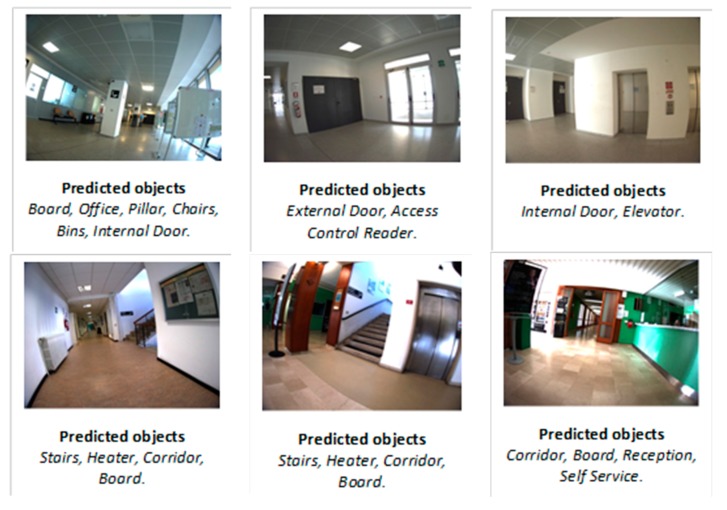
Example of results obtained by the proposed multilabeling fusion approach for both datasets. Upper row for Dataset 1, and lower one for Dataset 2.

The classification results of the fusion schemes are summarized in Table 2 and examples of results obtained for some query images are provided in Figure 9 for both datasets. As a first remark, it can be spotted that significant gains have been introduced with respect to using individual features (Table 1), which strengthens the assumption that fusing multiple features is likely to be advantageous over individual feature classification scenarios.

Another observation is that the average classification rate gradually increases from Fusion 1 all the way to Fusion 3, with minor disparities. Moreover, the first two strategies seem to favour the SPE over the SEN on both datasets, while Fusion 3, which performs fusion at decision level, favours SEN on Dataset 1 and exhibits a better SEN-SPE balance on Dataset 2. As a matter of fact, choosing between SEN or SPE depends upon the application being addressed. In our case, we will privilege SEN as we think it is more important to provide information on the presence of objects (even if it generates some false positives) rather than on the absence of objects. For such purpose, late fusion of individual decisions (i.e., Fusion 3) has proved to be a more efficient option than feature-level fusion (i.e., the first two schemes), with a tendency to score higher or equal SEN rates with respect to SPE.

For the sake of comparison of Fusion 3 strategy with state-of-the-art methods, we considered the contribution made in [15], namely the Semantic Similarity-based Compressed Sensing (SSCS) and the Euclidean Distance-based Compressed Sensing (EDCS) techniques, and also three different pretrained Convolutional Neural Networks (CNNs) which are ResNet [24], GoogLeNet [25] and VDCNs [26]. As shown in Table 3, for Dataset 1, our strategy outperforms largely the reference work in [15] with at least 10% of improvement and between 2% to 5% compared to the three pretrained CNNs. Moreover, our method offers the advantage of yielding far higher SEN. Both observations can be traced back to two considerations. On the one hand, the work put forth in [15] makes use of a small-sized dictionary of learning images to represent a given image by means of a compressive sensing-based approach, which might succeed in representing an image that has good matches in the dictionary but may fail when it comes to an (outlier) image that has no match in the dictionary. On the other hand, the proposed approach proceeds by extracting robust features capable to capture different variations across the images, followed by a customized feature learning step that furthers their discrimination capacity, which is ultimately reflected on higher classification rates as the results tell. The same observations apply for Dataset 2 as seen in Table 4.

An interesting fact to point out is that the size of the images has a direct influence on the processing time. Therefore, an option was to scale down the image resolution from full size (640 × 480), to its half (320 × 240), then (128 × 96), and finally (64 × 48), which respectively define a 100%, 50%, 20%, and 10% of the original size. The classification results in terms of AVG accuracy are shown in Table 5 and Table 6 for both datasets, respectively. It can be observed that the accuracy does not manifest drastic changes as the image size drops. In fact, there are instances where the smallest resolutions introduce slight improvements, which we believe can be interpreted by the fact that, in many images, there are large background surfaces (e.g., walls) that have usually uniform colours and textures, which may not be really useful as salient visual properties by which the images can be discriminated, reducing the image size thereby reduces the size occupied by those backgrounds, which may either maintain or even raise the classification performance.

Besides the classification rates, another important performance parameter is the runtime. For the proposed method, the runtime includes the feature extraction, the prediction and the fusion times. We provide the average processing time per image for both datasets in Table 7 and Table 8, from which it can be seen that, as expected, the runtime decreases with the image size, with our method being at least four times faster than the best runtime (GoogLeNet) provided by methods of reference. Particularly, 22 milliseconds per image is a very promising time span provided that fifteen objects are targeted in this work. Such processing time is based on a Matlab R2016b implementation, which is subject to be drastically reduced under for instance a C++ implementation. It is also worth mentioning that the number of objects in our work does not impact on the classification process.

## 6. Conclusions

This paper presented a scene description (via image multilabeling) methodology meant to assist visually impaired people to conceive a more accurate perception about their surrounding objects in indoor spaces. The idea of the proposed method is promoted around detecting multiple objects at once within a possible short runtime. A key-determinant of our image multi-labeling scheme is that the number objects is independent of the classification system, which entails the property of detecting as many objects as desired (depending on the offline setup to be customized by the user) within the same amount of time which amounts for much less than a second in our work.

The multi-labeling algorithm exploits feature learning concept by means of an autoencoder neural network, which amply demonstrated a significant potential in generating discriminative image representations.

Pros: In the literature, there exist several multi-object recognition methods (but not in the context of visually impaired rehabilitation). Those methods, may show interesting recognition efficiency, but they are dependent on the number of objects considered. By contrast, our method as hinted earlier, does not, which renders it much faster yet more reliable if considered in real-time scenarios. The earlier two points are technically verified in [15], where it was concluded that coarse image description is more adequate in this sense.

Cons: While the aim of the conducted coarse description is to roughly list the present objects as to bridge the gap between the real indoor setup and the image conceived in the visually disabled person’s imagination, inferring further information pertaining to the detected object’s location in the indoor space remains a vivid endeavour in our future considerations. This, however, may come at the cost of a heavier processing but it is not out of question. To complement this missing component, we suggest to find a way to introduce the depth information (e.g., through Kinect sensors for instance) as a post-operation. Another issue is related to the scalability of the system since it will need to be completely retrained in case the set of predefined objects requires to be modified quantitatively or qualitatively. As a future work, it is worth mentioning that the thresholding step could be made more sophisticated as proposed in [27].

## Figures and Tables

**Table 1:** Obtained recognition results using single features.

Dataset	Dataset 1			Dataset 2		
Method	HOG	BoW_RGB	LBP	HOG	BoW_RGB	LBP
SEN (%)	76.77	79.77	76.77	72.73	88.64	81.82
SPE (%)	82.16	82.9	80.07	88.67	90.24	86.27
AVG (%)	79.46	81.33	78.42	80.7	89.44	84.04

**Table 2:** Classification outcomes of all the fusion schemes.

Dataset	Dataset 1			Dataset 2		
Method	SEN (%)	SPE (%)	AVG (%)	SEN (%)	SPE (%)	AVG (%)
Fusion 1	79.40	87.45	83.42	85.00	91.80	88.40
Fusion 2	80.89	87.69	84.29	87.27	91.56	89.41
Fusion 3	89.51	81.30	85.40	90.00	90.12	90.06

**Table 3:** Comparison of classification rates on Dataset 1.

Method	SEN (%)	SPE (%)	AVG (%)
SSCS	79.77	66.54	73.15
EDCS	69.66	80.19	74.92
ResNet	66.29	94.46	80.38
GoogLeNet	67.04	94.22	80.63
VDCNs	71.91	94.46	83.19
Ours	89.51	81.3	85.40

**Table 4:** Comparison of classification rates on Dataset 2.

Method	SEN (%)	SPE (%)	AVG (%)
SSCS	75	74.09	74.54
EDCS	70	90.12	80.06
ResNet	68.18	96.75	82.46
GoogLeNet	72.27	97.11	84.69
VDCNs	81.82	96.39	89.10
Ours	90.00	90.12	90.06

**Table 5:** Comparison of classification rates on Dataset 1 under different resolutions.

Method	100%	50%	20%	10%
SSCS	73.15	73.34	74.52	74.51
EDCS	74.92	74.74	75.11	75.43
ResNet	80.38	79.88	79.32	78.76
GoogLeNet	80.63	81.63	82.52	79.02
VDCNs	83.19	83.37	84.50	84.57
Ours	85.40	86.02	86.14	86.63

**Table 6:** Comparison of classification rates on Dataset 2 under different resolutions.

Method	100%	50%	20%	10%
SSCS	74.54	74.54	73.91	74.48
EDCS	80.06	80.06	79.30	78.60
ResNet	82.46	82.40	84.69	87.13
GoogLeNet	84.69	84.69	84.74	84.12
VDCNs	89.10	88.71	87.80	88.13
Ours	90.06	90.34	90.03	90.69

**Table 7:** Comparison of average runtime per image on Dataset 1 under different resolutions.

Method	100%	50%	20%	10%
SSCS	2.16	1.42	1.22	1.17
EDCS	2.44	1.41	1.1	1.08
ResNet	0.136	0.132	0.131	0.131
GoogLeNet	0.100	0.098	0.096	0.093
VDCNs	0.300	0.295	0.291	0.288
Ours	1.230	0.200	0.048	0.022

**Table 8:** Comparison of average runtime per image on Dataset 2 under different resolutions.

Method	100%	50%	20%	10%
SSCS	2.66	1.53	1.21	1.17
EDCS	2.69	1.54	1.23	1.2
ResNet	0.136	0.132	0.131	0.131
GoogLeNet	0.100	0.098	0.096	0.093
VDCNs	0.300	0.295	0.291	0.288
Ours	1.230	0.200	0.048	0.022

## References

[1] Pundlik S., Tomasi M., Luo G. Collision Detection for Visually Impaired from a Body-Mounted Camera. Proceedings of the IEEE Conference on Computer Vision and Pattern Recognition Workshops.

[2] Balakrishnan G., Sainarayanan G., Nagarajan R., Yaacob S. Stereopsis method for visually impaired to identify obstacles based on distance. Proceedings of the 3rd International Conference on Image and Graphics (ICIG’04).

[3] Ulrich I., Borenstein J. (2001). The GuideCane-applying mobile robot technologies to assist the visually impaired. IEEE Trans. Syst. Man Cybern. Part Syst. Hum..

[4] Shoval S., Borenstein J., Koren Y. (1998). Auditory guidance with the Navbelt-a computerized travel aid for the blind. IEEE Trans. Syst. Man Cybern. Part C Appl. Rev..

[5] Bousbia-Salah M., Bettayeb M., Larbi A. (2011). A Navigation Aid for Blind People. J. Intell. Robot. Syst..

[6] Scalise L., Primiani V.M., Russo P., Shahu D., Mattia V.D., Leo A.D., Cerri G. (2012). Experimental Investigation of Electromagnetic Obstacle Detection for Visually Impaired Users: A Comparison with Ultrasonic Sensing. IEEE Trans. Instrum. Meas..

[7] López-De-Ipiña D., Lorido T., López U. BlindShopping: Enabling Accessible Shopping for Visually Impaired People through Mobile Technologies. Proceedings of the Toward Useful Services for Elderly and People with Disabilities.

[8] Pan H., Yi C., Tian Y. A primary travelling assistant system of bus detection and recognition for visually impaired people. Proceedings of the IEEE International Conference on Multimedia and Expo Workshops (ICMEW).

[9] Hasanuzzaman F.M., Yang X., Tian Y. (2012). Robust and Effective Component-Based Banknote Recognition for the Blind. IEEE Trans. Syst. Man Cybern. Part C Appl. Rev..

[10] Tang T.J.J., Lui W.L.D., Li W.H. Plane-based detection of staircases using inverse depth. Proceedings of the Australasian Conference on Robotics and Automation.

[11] Yang X., Tian Y. Robust door detection in unfamiliar environments by combining edge and corner features. Proceedings of the IEEE Computer Society Conference on Computer Vision and Pattern Recognition—Workshops.

[12] Wang S., Tian Y. Camera-Based Signage Detection and Recognition for Blind Persons. Proceedings of the 13th International Conference Computers Helping People with Special Needs.

[13] Mocanu B., Tapu R., Zaharia T. (2016). When Ultrasonic Sensors and Computer Vision Join Forces for Efficient Obstacle Detection and Recognition. Sensors.

[14] Mekhalfi M.L., Melgani F., Bazi Y., Alajlan N. (2015). Toward an assisted indoor scene perception for blind people with image multilabeling strategies. Expert Syst. Appl..

[15] Mekhalfi M.L., Melgani F., Bazi Y., Alajlan N. (2015). A Compressive Sensing Approach to Describe Indoor Scenes for Blind People. IEEE Trans. Circuits Syst. Video Technol..

[16] Dalal N., Triggs B. Histograms of oriented gradients for human detection. Proceedings of the IEEE Computer Society Conference on Computer Vision and Pattern Recognition (CVPR’05).

[17] Zhang Y., Jin R., Zhou Z.-H. (2010). Understanding bag-of-words model: A statistical framework. Int. J. Mach. Learn. Cybern..

[18] Zhang S., Yu X., Sui Y., Zhao S., Zhang L. (2015). Object Tracking with Multi-View Support Vector Machines. IEEE Trans. Multimed..

[19] Moranduzzo T., Melgani F. (2014). Detecting Cars in UAV Images with a Catalog-Based Approach. IEEE Trans. Geosci. Remote Sens..

[20] Ojala T., Pietikainen M., Maenpaa T. (2002). Multiresolution gray-scale and rotation invariant texture classification with local binary patterns. IEEE Trans. Pattern Anal. Mach. Intell..

[21] Guo Z., Zhang L., Zhang D. (2010). A Completed Modeling of Local Binary Pattern Operator for Texture Classification. IEEE Trans. Image Process..

[22] Vincent P., Larochelle H., Lajoie I., Bengio Y., Manzagol P.-A. (2010). Stacked Denoising Autoencoders: Learning Useful Representations in a Deep Network with a Local Denoising Criterion. J. Mach. Learn. Res..

[23] Rumelhart D.E., Hinton G.E., Williams R.J. (1986). Learning representations by back-propagating errors. Nature.

[24] He K., Zhang X., Ren S., Sun J. Deep Residual Learning for Image Recognition. Proceedings of the IEEE Conference on Computer Vision and Pattern Recognition (CVPR).

[25] Szegedy C., Liu W., Jia Y., Sermanet P., Reed S., Anguelov D., Erhan D., Vanhoucke V., Rabinovich A. Going deeper with convolutions. Proceedings of the IEEE Conference on Computer Vision and Pattern Recognition (CVPR).

[26] Simonyan K., Zisserman A. (2014). Very Deep Convolutional Networks for Large-Scale Image Recognition. arXiv.

[27] Zeggada A., Melgani F., Bazi Y. (2017). A Deep Learning Approach to UAV Image Multilabeling. IEEE Geosci. Remote Sens. Lett..

